# Combined Effects of Admission Serum Creatinine Concentration with Age and Gender on the Prognostic Significance of Subjects with Acute ST-Elevation Myocardial Infarction in China

**DOI:** 10.1371/journal.pone.0108986

**Published:** 2014-10-10

**Authors:** Zhao-Yang Li, Pu-Liu, Zhao-Hong Chen, Feng-Hui An, Li-Hua Li, Li-Li, Chang-Yan Guo, Yan Gu, Zhe Liu, Tie-Bing Zhu, Lian-Sheng Wang, Chun-Jian Li, Xiang-Qing Kong, Wen-Zhu Ma, Zhi-Jian Yang, En-Zhi Jia

**Affiliations:** 1 Department of cardiovascular medicine, the first affiliated hospital of Nanjing Medical University, Nanjing, Jiangsu Province, China; 2 Department of Cardiovascular Medicine, the Friendship Hospital of Ili Kazakh Autonomous Prefecture, Yining, XinJiang, China; Temple University School of Medicine, United States of America

## Abstract

**Objective:**

to explore the impact of admission serum creatinine concentration on the in-hospital mortality and its interaction with age and gender in patients with acute ST-segment elevation myocardial infarction (STEMI) in China.

**Methods:**

1424 acute STEMI patients were enrolled in the study. Anthropometric and laboratory measurements were collected from every patient. A Cox proportional hazards regression model was used to determine the relationships between the admission serum creatinine level (Cr level), age, sex and the in-hospital mortality. A crossover analysis and a stratified analysis were used to determine the combined impact of Cr levels with age and gender.

**Results:**

Female (HR 1.687, 95%CI 1.051∼2.708), elevated Cr level (HR 5.922, 95%CI 3.780∼9,279) and old age (1.692, 95%CI 1.402∼2.403) were associated with a high risk of death respectively. After adjusting for other confounders, the renal dysfunction was still independently associated with a higher risk of death (HR 2.48, 95% CI 1.32∼4.63), while female gender (HR 1.19, 95%CI 0.62∼2.29) and old age (HR 1.77, 95%CI 0.92∼3.37) was not. In addition, crossover analysis revealed synergistic effects between elevated Cr level and female gender (SI = 3.01, SIM = 2.10, AP = 0.55). Stratified analysis showed that the impact of renal dysfunction on in-hospital mortality was more pronounced in patients <60 years old (odds ratios 11.10, 95% CI 3.72 to 33.14) compared with patients 60 to 74 years old (odds ratios 5.18, 95% CI 2.48∼10.83) and patients ≥75years old (odds ratios 3.99, 95% CI 1.89 to 8.42).

**Conclusion:**

Serum Cr concentration on admission was a strong predictor for in-hospital mortality among Chinese acute STEMI patients especially in the young and the female.

## Introduction

ST-segment elevation myocardial infarction (STEMI) is a clinical syndrome defined by characteristic symptoms of myocardial ischemia in association with persistent electrocardiographic (ECG) ST elevation and subsequent release of biomarkers of myocardial necrosis [1]. Worldwide, coronary artery disease (CAD) is the single most frequent cause of death. Over seven million people every year die from CAD, accounting for 11.2% of all deaths [2]. Differing from USA, where the incidence of acute STEMI and rates of death attributable to CVD have declined [3], [4], cardiovascular diseases are rising as the primary cause of death and disability in China in decades [5], [6]. Patients with acute STEMI face higher in-hospital mortality for many factors. Renal dysfunction has been established as one of the most important predictors of in-hospital and long-term mortality in the acute STEMI patients [7], [8], Moreover, age as another significant predictor of adverse outcomes in this group, is related with the renal function [9]–[11], It has also been reported that females with acute myocardial infarction (AMI) have a higher risk of adverse events [12], [13]. Nevertheless, the relationship between the admission serum creatinine concentration (µmol/l) and the prognosis of acute STEMI patients has not been well characterized in China and few data are available concerning its interaction with age and gender. So, in our study, we aimed to examine the impact of admission serum creatinine concentration on the in-hospital mortality and its interaction with age and gender in patients with STEMI in China.

## Materials and Methods

### Study subjects

The patients admitted to First Affiliated Hospital of Nanjing Medical University, Nanjing (China) for acute STEMI from 1 January 2003 to 31 December 2010 (n = 1467) were enrolled in the study. Among these, 43 patients were excluded due to missing data of admission serum creatinine concentration, leaving 1424 patients (296 females), aging 26 to 98 years old for the analysis. All patients had no history of chronic renal diseases or tumors. The current guidelines for the ECG diagnosis of the STEMI require at least 1 mm (0.1 mV) of ST segment elevation in the limb leads, and at least 2 mm elevation in the precordial leads [14]. Coronary angiograms were scored according to Gensini's score. According to this system, a substantial reduction in lumen diameter is assigned a higher score than a distal lesion [15]. The primary outcome of the study was death from all causes during hospitalization. The study was approved by the Ethics Committee of the First Affiliated Hospital of Nanjing Medical University, and written informed consent was obtained from each patient.

### Blood sample and biochemistry measurements

The blood was withdrawn in the emergency room or coronary care unit within 12 hours since admission from every patient. All measurements were conducted at the clinical laboratory in the First Affiliated Hospital of Nanjing Medical University. The creatinine (Cr, µmol/L) levels were determined by enzymatic procedures on an automated auto-analyzer (AU 2700 Olympus, First Chemical Ltd, Tokyo, Japan).

### Statistical analysis

Data were statistically analyzed using Statistics Package for Social Sciences (ver. 16.0; SPSS Incorporated, Chicago, IL, USA). Subjects were classified into 2 groups according to gender. Skewed distributed parameters were presented as medians with interquartile ranges and comparisons were analyzed by the Mann-Whitney U test. Categorical variables were compared between groups by chi-squared analysis. If 1of the cell expected counts has value less than 10, we used Fisher's exact test instead of chi-squared test to obtain P values for categorical variables. Receiver-operating characteristic (*ROC*) curve analysis was used to determine the optimum cut-off points of age and admission serum Cr concentration best predicting adverse outcome in this group. Hazard ratios (HR) and 95% confidence intervals (CI) for all-cause mortality were calculated using a *Cox* proportional hazards regression model. Differences were considered to be significant if the null hypothesis could be rejected with >95% confidence. All *p*-values are two-tailed.

To analyze possible positive or negative interactions between admission Cr levels and age or sex, 4×2 table approach was used to calculate Hazard ratios (HR), respective 95% confidence intervals (CI) and two-tailed *p* values, as well as synergy measures in additive (SI) and multiplicative models (SIM). It was assumed that unexposed individuals without the risk creatinine level have a certain background risk for all-cause mortality (HR_00_ is assumed to be 1); HR_10_ refers to the relative risk for all-cause mortality among people without the risk creatinine level but exposed to the environmental risk factor (old age or female gender)relative to those with neither the risk creatinine level nor exposure; HR_01_ refers to the relative risk among people with the risk creatinine level who are not exposed to the risk factors relative to those with neither the risk creatinine level nor exposure; HR_11_ is the ratio of all-cause mortality risk among exposed people with risk creatinine level relative to unexposed people without the renal dysfunction. These HRs were then used in the calculation of synergy indexes: SI = (HR_11_-1)/(HR_10_+HR_01_-2), SIM = HR_11_/(HR_10_×HR_01_); the relative excess risk due to interaction, RERI = HR_11_-HR_10_-HR_01_+1;and the attributable proportion of the disease due to interaction, AP = RERI/HR_11_ [12], [13]. We then divided patients into three groups on the basis of age: <60 years old, 60∼74 years old, ≥75 years old. Odds ratios and 95% CIs were calculated for patients with elevated Cr level compared with patients with normal Cr level in each age group to examine the combined impact of age and serum Cr level on in-hospital mortality.

## Results

### Clinical characteristics of study population

The clinical characteristics of patients are shown in **Table 1**. A total of 1424 patients (1128 males and 296 females; median age, 65 years (range, 26–98 years)) were enrolled in the study. Female patients were older (p<0.001), less smoking and drinking (p<0.001), getting more diabetes (p = 0.007), having lower serum Cr level (p<0.001), getting less stent use (p<0.001) and having higher in-hospital mortality (p = 0.01), but no differences were found in the location of MI (p>0.05), history of hypertension (p = 0.32) and stroke (p = 0.38), EF of LV (p = 0.13) and Gensini score (p = 0.37) across two groups.

**Table 1:** Clinical characteristics of male and female patients with STEMI.

	Male (n = 1128)	Female (n = 296)	P value
Age (years)	61(52–71)	67(60–74)	<0.001
Smoking (%)	621(55.1%)	18(6.08%)	<0.001
Drinking (%)	272(24.1%)	4(1.35%)	<0.001
Hypertension (%)	619(54.8%)	172(58.1%)	0.32
Diabetes (%)	251(13.4%)	88(51.2%)	0.007
Stroke (%)	50(4.43%)	16(5.40%)	0.38
Location of MI			
Anterior (%)	332(29.4%)	98(33.1%)	0.22
Inferior (%)	454(40.2%)	111(37.5%)	0.39
Lateral (%)	39(3.45%)	9(3.04%)	0.72
CK level (U/L)	346(87.9–1673)	202(64.3–817)	<0.001
Cr level (µmol/L)	79.0(67.9–91.45)	63.5(56.0–74.6)	<0.001
EF of LV (%)	61.8(56.0–65.3)	62.9(58.5–66.4)	0.13
Gensini score	53.0(30.5–83.0)	50.0(31.0–80.5)	0.37
Stent use (%)	759(67.3%)	149(50.3%)	<0.001
Mortality (%)	56(4.96%)	26(8.78%)	0.01

CK: Creatinine kinase; Cr: Creatinine; EF of LV: Ejection fraction of left ventricular.

### Results of ROC curve analysis and cox proportional hazards analysis

**Figure 1:**
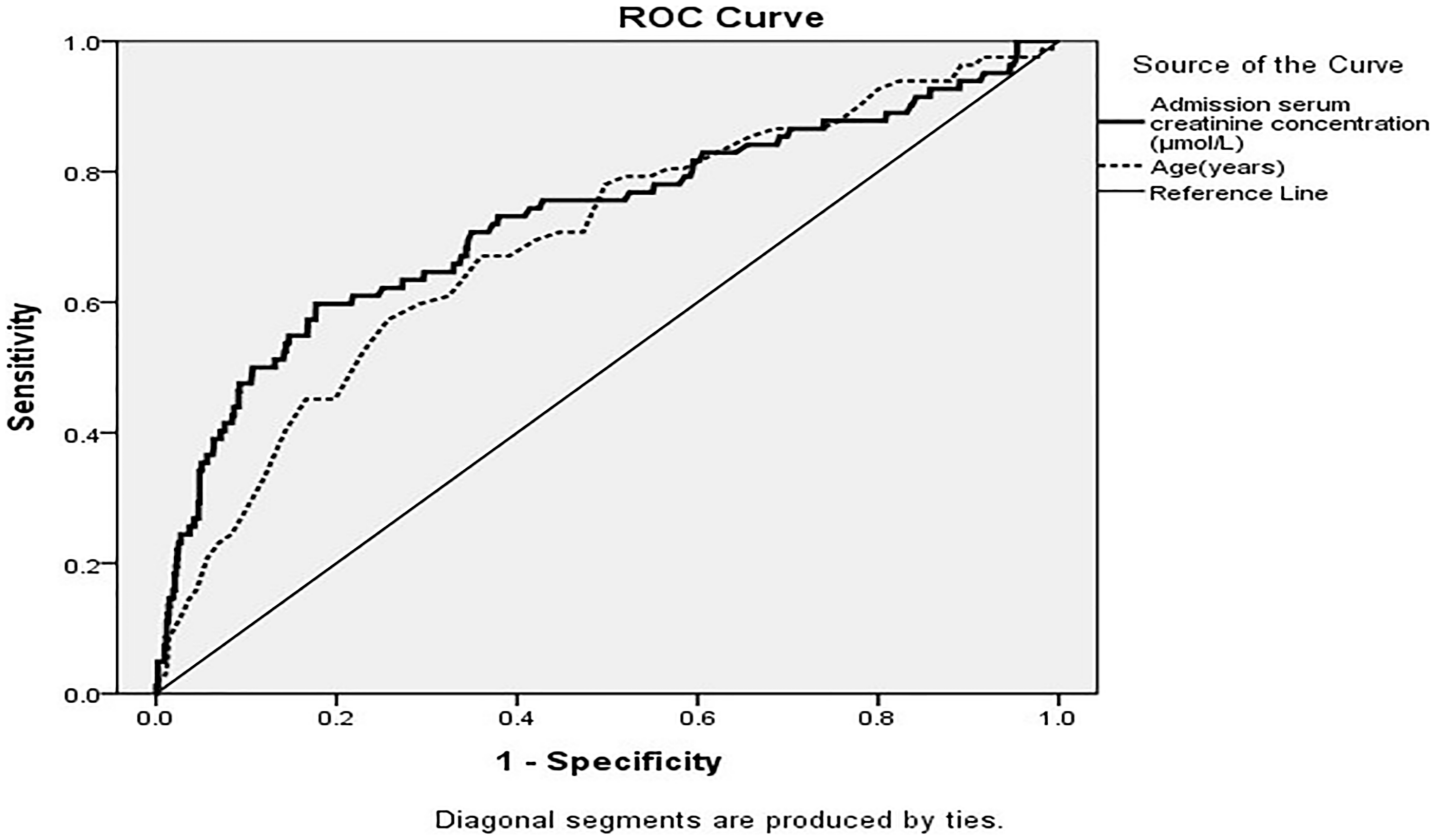
Receive operating characteristic analysis of age(years) and admission serum Cr level(µmol/l) in predicting all-cause mortality.

Receiver operating characteristic (ROC) analysis was applied to demonstrate cut-off points of serum creatinine 99.30 µmol/l and 73 year-old best predicting in-hospital mortality respectively. So, the normal serum creatinine group (normal group) was defined as <99.30 µmol/l, and the elevated serum creatinine group (elevated group) was defined as ≥99.30 µmol/l. while the young group <73 years old and the old group ≥73 years old. The results of ROC curve analysis were showed in **Table 2** and **Figure 1**.Univariate Cox proportional hazards regression model showed old age (HR 1.69, 95%CI 1.40∼2.04), female gender (HR 1.69, 95%CI 1.05∼2.71), and renal dysfunction (HR 5.92, 95%CI 3.78∼9.28) were all associated with a higher in-hospital mortality. After adjusting for other confounders, including smoking and drinking status, diabetes, CK levels and stent use, the renal dysfunction was still strongly associated with a higher risk of death (HR 2.48, 95% CI 1.32∼4.63), while female gender(HR 1.19,95%CI 0.62∼2.29) and old age (HR 1.77, 95%CI 0.92∼3.37) not (**Table 3**).

**Table 2:** Receive operating characteristic analysis of age(years) and admission serum creatinine level (µmol/l) in predicting all-cause mortality.

*Variable*	*Cut-off*	*Sensitivity*	*Specificity*	*AUC*	*95%CI*	*P*
Age(years)	73	0.57	0.77	0.69	(0.63∼0.75)	<0.001
Creatinine(µmol/l)	99.30	0.60	0.82	0.73	(0.66∼0.79)	<0.001

AUC: Area under the curve; 95%CI: 95% confidence interval.

**Table 3:** Cox proportional hazards regression analysis of the effect of risk factors on in-hospital mortality.

*Variable*	*B*	*SE*	*Wald*	*hazard ratios (95% CI)*	*P value*	*Adjusted HRs(95%CI)*
Age(old/young)	1.20	0.23	28.0	3.33(2.13–5.20)	<0.001	1.77(0.92–3.37)
Gender (female/male)	0.52	0.24	4.69	1.69(1.05–2.71)	0.03	1.19(0.62–2.29)
Creatinine (elevated/normal)	1.78	0.23	60.3	5.92(3.78–9.22)	<0.001	2.48(1.32–4.63)

### Interaction between Cr level and age or sex

**Figure 2:**
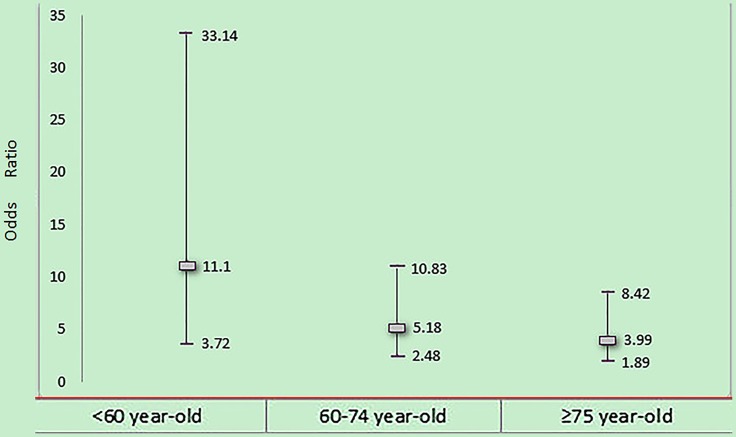
Odds ratios and 95% CIs of in-hospital mortality in 3 age groups: young (<60 years old), middle-aged (60∼74 years old) and elderly (≥75years old) according to admission serum Cr level compared with patients with normal renal function. p<0.01 by Cochran's and Mantel-Haenszel tests.

The analysis of the possible positive/negative association between renal dysfunction and old age or female gender is expressed in **Table 4** and **Table 5**. Regarding the conventional risks of the male or young with normal Cr level (reference category) as being 1.0, the HRs estimating the effects of joint exposure to elevated Cr level and female gender or old age were significantly higher than the HRs estimating the effects of each factor in the absence of the other. The risk provided by renal dysfunction was found to be positively reinforced by the female gender (SI = 3.01, SIM = 2.10, AP = 0.55) for in-hospital morality in the acute STEMI patients. Since the synergy index in multiplicative model (SIM) for renal dysfunction and old age was less than 1 (SI = 1.34, SIM = 0.57, AP = 0.14), we then performed a stratified analysis. Patients were divided into three groups stated above. As expected, the old patients had higher mortality. But, the adjusted risk of in-hospital mortality for patients with renal insufficiency compared with those with normal renal function was higher in young patients (odds ratio 11.10, 95% CI 3.72 to 33.14) than middle-aged patients (odds ratio 5.18, 95% CI 2.48∼10.83) and old patients (odds ratio 3.99, 95% CI 1.89 to 8.42) (**Table 6** and **Figure 2**).

**Table 4:** Combined effect of admission serum Creatinine level and age in predicting all-cause mortality.

*Risk Age*	*Risk Creatinine*	*Death*	*Alive*	*HR(95%CI)*
0	0	18	873	1
0	1	17	124	6.33(3.23–12.4)
1	0	15	231	2.96(1.48–5.94)
1	1	32	114	10.8(5.95–19.5)

SI = 1.34;SIM = 0.57;RERI = 1.49;AP = 0.14.

Zero refers to the young or normal Cr group while one the old or elevated group.

**Table 5:** Combined effect of admission serum Creatinine level and gender in predicting all-cause mortality.

*Risk Gender*	*Risk Creatinine*	*Death*	*Alive*	*HR(95%CI)*
0	0	23	868	1
0	1	33	204	4.99(2.92–8.56)
1	0	10	236	1.34(0.62–2.89)
1	1	16	34	14.0(7.41–26.6)

SI = 3.01;SIM = 2.10;RERI = 7.71;AP = 0.55.

Zero refers to the male or normal Cr group while one the female or elevated group.

**Table 6:** Unadjusted mortalities of each age group across different serum Cr level.

Creatinine levels(µmol/L)	Age groups(years)			P value
	<60(n = 522)	60∼74(n = 599)	≥75(n = 303)	
Normal Cr level	1.5%(7/473)	3.1%(15/486)	6.2%(11/178)	0.01
Elevated Cr level	14.3%(7/49)	14.2%(16/113)	20.8%(26/125)	0.34

## Discussion

In this study, we showed that admission serum Cr level was a strong predictor for in-hospital mortality among acute STEMI patients in China and had combined impacts with age and gender.

As early as in 1998, Herzog et al. had unveiled that patients with end-stage renal disease suffering acute MI faced the horrible prognosis [16].Recently, In Yamaguchi J et al.'s study, the mortality of in-hospital and long-term in patients with AMI, even undergoing successful primary PCI, was greater when serum Cr level was elevated even slightly [17], [18]. The concept of renal dysfunction as a potent risk factor for the adverse outcomes in patients with cardiovascular disease has attracted a great deal of attention. There are multiple possible explanations for the association of higher mortality and elevated serum Cr level, such as serum creatinine was a marker of specific vascular disease, chronic overload, endothelial dysfunction, impaired myocardial blood flow [7], [19], [20]. It has been reported that hypoperfusion after the acute STEMI, nephrotoxicity of contrast medium used for primary PCI and other cardiovascular drugs could induce the renal dysfunction [11], [21]. And the renal dysfunction is associated with adverse LV remodeling indicating combined cardiac and renal dysfunction (called “cardio-renal syndrome”), which synergistically accelerates the progression of startling morbidity and mortality [21], [22].

In addition, Clinical studies have greatly expanded efforts to recognize the importance of gender and age in the prognosis of acute STEMI patients [9], [13], [23], [24], revealing female gender and old age as predictors of adverse outcomes in patients with acute STEMI.

The higher mortality for the old is not surprising given the high prevalence of risk factors and graver heart condition in these patients. In our research, after adjusting for related confounders described above, old age was no more a significant predictor for in-hospital mortality in acute STEMI patients. Previous studies has clarified that advanced age is associated with narrowing and blunted vasodilatory capacity of renal vessels inducing reduced renal perfusion and impairing renal function, which lead to adverse LV remodeling stated above [11],[22]. Though mortality was higher in the old, the Odds ratio and 95%CI were greater in the young, meaning that the impact of worsening renal function appears to be greater in young patients compared with middle-aged and old patients. Similar paradox was found in Cardarelli F et al.'s observation [25].

Cr level of the female was relatively lower compared with men (female patients (median age, 70) much older than the male patients (median age, 63)) in this study (p<0.001). This may partially attribute to the lower muscle mass of women. Thus, the same Cr level may indicate worse renal damage in female patients. Also, a higher prevalence of chronic renal insufficiency (CRI) had been confirmed in DM2 patients [26], and women with diabetes have more than a 40% greater risk of incident coronary heart disease compared with men with diabetes [27]. So, we consider the higher prevalence of diabetes in female patients also may account for the synergistic effect of renal dysfunction and female gender on in-hospital mortality in this study. Other age-related risk factors and co-morbid conditions could be associated with the high in-hospital mortality in female patients as well [13]. Similarly, the higher mortality of female patients may due to the older age, more diabetes and less stent use in our research. Our data showed synergistic effect of renal dysfunction and female gender on in-hospital mortality in patients with acute STEMI. The mechanism of the phenomenon needs more studies.

These observations unveil the horrible prognosis faced by acute STEMI patients with elevated admission serum Cr concentration, especially by the young and female patients, calling for targeted strategies to reduce the burden of risk in this population. And future clinical trials studying therapies for coronary artery disease need to be more inclusive of patients with renal insufficiency [28].

## Limitations

There were also a few limitations to our study. First, the study was single-centered and the number of the cases was limited. Second, using serum creatinine concentration to measure the renal function may underestimate its risk on the basis that it has been clarified that glomerular filtration rate estimates renal function more precisely [25].

## Conclusions

In this study, we showed that serum Cr concentration on admission was a strong predictor for in-hospital mortality in patients with acute STEMI in China in association with age and gender.
